# Digital activism in Southeast Asia: the #MilkTeaAlliance and prospects for social resistance

**DOI:** 10.3389/fsoc.2024.1478630

**Published:** 2024-11-25

**Authors:** Bama Andika Putra

**Affiliations:** ^1^School of Sociology, Politics, and International Studies, University of Bristol, Bristol, United Kingdom; ^2^Department of International Relations, Universitas Hasanuddin, Makassar, Indonesia

**Keywords:** digital activism, Southeast Asia, Myanmar, Milk Tea Alliance, online platform

## Abstract

Digital activism in Southeast Asia is on the rise. The Milk Tea Alliance, known as a collaboration of netizens voicing human rights concerns in Asia, has now reached the citizens of Southeast Asian autocratic regimes, including Myanmar. Having faced decades of human rights oppression and undemocratic rule, the Milk Tea Alliance of Myanmar has been vocal in disseminating the post-2021 military coup domestic situation and reimagining what a democratic Myanmar would consist of in the future. This perspective article argues the significance of digital activism for Myanmar by tracing what the existing literature misses in assessing this phenomenon and the nexus between Myanmar’s Milk Tea Alliance and the prospects of change in the state’s democratic landscape. Taking into consideration the development of digital activism in Myanmar between 2020 and 2024 and adopting the theoretical frameworks of “legislative opportunism” and “liberation technology,” this study concludes several possibilities: (1) the massive following of Myanmar’s Milk Tea Alliance could establish the foundations for offline movements mimicking the Arab Spring; (2) the harnessing of democratic thoughts among citizens; and (3) changes from within are feasible through digital activism compared to externally imposed actions such as through the regional organization of Southeast Asia, ASEAN.

## 1 Digital activism in Southeast Asia: what the existing literature is missing

The presence of undemocratic regimes in Southeast Asia is concerning. Thailand’s 2014 coup and the Tatmadaw’s (Myanmar military) unilateral decision to take over Myanmar’s government on February 1, 2021, marks how fragile this region is towards authoritarian rule. Although it would be simple to predict the downfall of these nations due to domestic instability, this perspective article attempts to analyze the “taken for granted” development in those regions, namely, the rise of digital activism. With a massive online presence, Southeast Asia’s digital activism potential is endless. As Strangjo assessed, “the internet and social media’s arrival in the region have led to unprecedented levels of grassroots activism across societies” (Strangjo, 2020).

Discussions on Myanmar’s digital activism since the 2021 coup are intriguing. Fighting back against tyranny, Myanmar’s youth have taken both the streets and online platforms to counter repressions known as the “spring revolution” (Palatino, 2024). In digital platforms, the people of Myanmar netizens have taken the initiative to join the #MilkTeaAlliance, an online pro-democracy movement echoing domestic change and reporting instances of human rights oppression online (Bunyavejchewin, 2020; Duangdee, 2021; Lee, 2021). Through this, Myanmar netizens hope to garner sympathy, aiming to spread the news about what is truly happening within its borders. The Milk Tea Alliance is a multinational protest movement that started as an internet meme countering Chinese nationalist comments on vast social media platforms (McDevitt, 2020; Tanakasempipat, 2020). Chinese national comments arose due to the widely covered news on the South China Sea and tensions between Chinese and Taiwanese supporters (Fravel, 2011; Putra, 2020, 2023; Putra and Cangara, 2022; Reuters, 2023). The initial intentions of the movement are to counter Chinese comments and ensure that people accessing social media worldwide can be provided real-time information on the crisis from alternative perspectives. Now acting as a symbol for resisting authoritarian rule, this online movement is active in Southeast Asia, involving Thailand, Myanmar, the Philippines, and Indonesia.

How has the existing literature made sense of this development? Unfortunately, few studies have assessed the nature of online movements in Myanmar or their future trajectories. The Milk Tea Alliance is not new; thus, it has generated a number of studies arguing this unique online political movement in Southeast Asia. Despite differences, they are simultaneously skeptical of its effectiveness due to the referenced government measures to counter online freedom of speech (Lim, 2023; Kreutz and Makrogianni, 2024). This comes with the realization that the number of citizens accessing online platforms in Southeast Asia is on the rise, with an estimated 68% having access to social media (Smith and Perry, 2022; Newman et al., 2023). In a specific study assessing how long youth spend on these online platforms, Kemp concluded that 16-24-year-olds spend an average of 10 h daily (Kemp, 2021). Consequently, the Southeast Asian government’s fear of fake news and disinformation spreading has been on the rise (Putra, 2024). The policy repercussions are what scholars have labeled as “legislative opportunism,” the hijacking of the digital environment by government stakeholders by imposing arbitrary censorships, especially in non-democratic countries of Southeast Asia (Sombatpoonsiri and Luong, 2022).

The nexus between Myanmar and the Milk Tea Alliance could benefit from an independent investigation. In its current state, scholars have argued for the importance of tracing the origins of the online movement’s norm in Myanmar (Kong, 2024) and assessing the nature of Myanmar’s “covert resistance” (Chan, 2024). This perspective article aims to contribute to this limited number of studies on Myanmar’s digital activism by arguing the prospects of Myanmar netizens’ Milk Tea Alliance movement towards permanent change within Myanmar. It argues that the dominant framework pushing the regional organization of ASEAN (Association of Southeast Asian Nations) is heavily incompatible as a conflict resolution mechanism and that only changes within can establish solid grounds for democracy. In doing so, this qualitative descriptive study utilizes secondary data between 2021 and 2024 and generates several possibilities concerning Myanmar’s democratic environment. It aims to investigate case studies and adopts two theoretical frameworks to assess the dynamics in those studies: “legislative opportunism” and “liberation technology.” While legislative opportunism is the process of digital environments hijacked by government stakeholders for their interests, liberation technology denotes the utilization of information and communication technology to foster political mobilization, such as anti-government movements (Morozov, 2011; Christensen, 2012; Manacorda and Tesei, 2020).

## 2 The #MilkTeaAlliance in Myanmar

The Milk Tea Alliance of Southeast Asia inspires digital activism in Myanmar. As a transnational collaborative effort to bring about change toward democracy, there is an understanding among those in the movement that countering tyranny matters most (Palatino, 2024). Furthermore, the nature of this informal network, which comprises pro-democracy digital activists across Asia, allows for a stronger bond of support established among the populations currently facing authoritarian rule. As the framework of liberal technology argues, unified action through the digital platform allows rapid information sharing to generate trending topics and memes, ultimately leading to political action due to the presence of popular beliefs constructed through digital platforms such as Facebook, X (formerly Twitter), etc.

Unlike traditional pro-democracy movements, Myanmar’s digital activism perceives the importance of both online and offline grassroots movements. Grassroots movements are typically conducted offline, taking the streets to show the people’s disapproval of government policies. However, in the case of the Milk Tea Alliance, the virtual nature of the protests generates questions on how far of a change this movement could lead for the nation. Seeing the history of the Milk Tea Alliance, a decisive impact of the movements in the construction of discourses within society. From what started with a Thai actor’s tweet stating that Hong Kong is a country being attacked by pro-Chinese internet trolls, an alliance of Taiwan, Hong Kong, and Thailand internet users re-established the discourse of freedom of speech in online platforms (Chia and Singer, 2021). A series of digital activism in Vietnam and Thailand challenging authoritarian legitimacy also constructs critical discourses of democracy in places of extremely limited rights (Strangjo, 2020).

In a conflict-torn state such as Myanmar, the use of social media to conduct “silent strikes” is effective in countering the risk of being arrested by the Tatmadaw. Myanmar is not a country that is conducive to carrying out peaceful protests. Since the coup in 2021, the Institute for Strategy and Policy Myanmar estimated that 8,640 citizens were killed (ISP, 2023). This high number is due to attacks by the Tatmadaw targeting protests and those victims of the ongoing war between insurgents and Myanmar’s military forces (Xinhua, 2021; APHR, 2023). The military junta’s legislative opportunism has also been in the form of forming barriers to access the internet, with 291 internet shutdowns taking place since the coup (Bradley, 2024). Justice is practically absent in Myanmar, as the coup also involved the arrest of high-level officials and political party members who were democratically elected in 2020. To better picture this situation to international audiences, Myanmar youth’s digital activism has documented these conditions to the best of their abilities, from empty streets to horrific actions in various parts of the nation (Palatino, 2024). However, a central role that this perspective article highlights is the discourses that it makes dominant. As Chia and Singer observed as the benefit of Milk Tea Alliance in X for Myanmar, “…a central force in shaping the way Myanmar’s youth understand the current battle between pro-democracy protestors and their vastly better-armed opponents, a predicament faced by other youth in neighboring countries” (Chia and Singer, 2021).

To counter the legislative opportunism shown by the military junta, a series of draconian measures and strict measures imposed on online digital platforms since the COVID-19 pandemic in Myanmar have built resilience within the youth. Understanding the difficulty of accessing social media and post-sensitive topics, Myanmar’s Milk Tea Alliance has used Virtual Private Networks (VPNs) to mask their identities and locations, making them untraceable by the Tatmadaw (Lee, 2021). This has first allowed for greater exposure to the country’s domestic situation by using trending hashtags such as #WhatsHappeningInMyanmar (NIKKEI, 2022; Phattharathanasut, 2024). Uniquely, this digital activism has evolved to have a common stance on what Myanmar netizens perceive as a proper democratic policy, which includes the stance of recognizing the democratically-elected political party, the National Unity Government of Myanmar (NUG), as the legitimate leaders of Myanmar (McDevitt, 2020; Chia and Singer, 2021; Chan, 2024; Kong, 2024). Pressing matters in the past, such as the status of the heavily prosecuted Rohingya people, has also led to the stance of the need to acknowledge these minorities for the sake of their protection in the future (Chia and Singer, 2021).

## 3 The future in the making: digital activism and domestic changes in Myanmar

What are the prospects of change in the context of Myanmar’s struggle for democracy and digital activism? This section argues the massive following could establish the foundations for offline movements mimicking the Arab Spring, the harnessing of democratic thoughts among citizens, and that changes from within are feasible through digital activism compared to externally imposed actions such as through ASEAN. The threefold arguments presented allow the author to conclude the impact of digital activism in Myanmar and how it potentially would shape the democratic landscape in the future.

The number of followers and those participating in the Milk Tea Alliance is considerably high. In a recent study, Kong concluded that 25 million hashtags were used on Twitter, with approximately 600.000 people posting about what was happening in Myanmar between 2020 and 2021 (Kong, 2024). One of the benefits of digital activism is its widespread coverage of information sharing among those on a similar platform. With the presence of social media, individuals can post information and updates on events and share videos through online platforms such as Facebook, YouTube, and TikTok; the call to action could not be more immediate. Scholars echoing the presence of liberation technology also argue the presence of more traditional platforms, such as blogs and the making of websites that have also been seen in Myanmar (Morozov, 2011; Christensen, 2012). In present times, locals already utilize social media as a call of action platform, thus synergizing online and offline protests (McDevitt, 2020; Chia and Singer, 2021; Chan, 2024; Kong, 2024). The only reason more extensive action has not occurred amid such rapid information is the ongoing armed conflict between the insurgencies and Tatmadaw.

The Milk Tea Alliance’s digital activism could lead to two scenarios in the current status quo. It could first lead to a more decisive response by global actors due to the violations of human rights perpetrated by the Myanmar military, or in contrast, not cause much change, such as in the case of Thailand. An inquiry is needed to assess the nexus between digital activism and global actions in international organizations such as the United Nations. On March 10, 2021, the United Nations Security Council unanimously agreed to condemn the Tatmadaw’s military actions (Lee, 2021). Thus, the stronger the presence of digital activism in Myanmar, with the daily reporting of events taking place and picturizing the crimes against humanity perpetrated, this potentially garnishes greater international support for action. However, this could also lead to what is happening with Thailand’s Milk Teal Alliance. Its movements, lasting for approximately the same years as Myanmar’s, have not been able to generate significant change towards the existing monarchy.

Despite the possibilities of success and failure, this perspective article argues the potential of change due to what digital activism led to in the Middle East’s Arab Spring. The Arab Spring is a series of armed rebellions in several Middle Eastern countries as a call to action against the undemocratic rule of Arab states. What started in Tunisia in the 2010s quickly reached neighboring countries of Bahrain, Libya, Yemen, Syria, and Egypt (Lawrence, 2017; Moore-Gilbert, 2018; Greijdanus et al., 2020; Chiovaro et al., 2021; Josua and Edel, 2021). Digital activism was influential in spreading the news to update the situation on the ground and to mobilize the masses for demonstrations on the street (Wolfsfeld et al., 2013; Smidi and Shahin, 2017; Sinpeng, 2019). This led to the deposed rules of Tunisia, Libya, and Egypt and established higher awareness of the importance of economic prosperity and human rights across the Middle East. The two conditions that influenced the Arab Spring, economic stagnation and the absence of human rights, are also present in the context of Myanmar. Although the social and political contexts may differ between the Middle East and Southeast Asia, the events of changes perpetrated by digital activism in the Middle East should be able to inspire the limitless impact that the Milk Tea Alliance could generate in their call for greater democracy in Myanmar. However, it is not the intention of this article to argue that the online movements seen in Myanmar will cause direct changes to the Myanmar political landscape. Taking the case of the 2008 Saffron Revolution and the presence of Myanmar activists using blogs to express concerns, it took 2 years until the military junta was willing to conduct Myanmar’s first general election in 20 years (Chowdhury, 2008; Selth, 2008).

The harnessing of democratic thoughts and foundations thus serves as the second possibility. In constructing this argument, we return to the dynamics of the Milk Tea Alliance in Myanmar and how it is consistent with the liberation technology arguments in the past. First, connections have been made between the online movement and the 2020 democratically-elected political party of the NUG, establishing the foundations of how digital activism is a platform to express opinions regarding taken policies within the state (Chia and Singer, 2021). Second, the transnational nature of Myanmar’s digital activism allows them to connect with like-minded individuals, establish links with activists in Asia, and share instances of government abuses with one another (Lee, 2021). And third, there have been more profound talks about democracy within this online platform on the direction of Myanmar’s democracy. Discussions on what aspects and regulations should be included in a new constitution and ways to establish a federal democracy are examples of constructive discussions to reinterpret what a future “democracy” in Myanmar would look like (Chia and Singer, 2021).

For a country that has been under constant military rule by the Tatmadaw, this could be pivotal in harnessing the democratic thoughts of the Myanmar youth. Having experienced undemocratic justice and rule for decades, the presence of human rights oppressions towards minorities, and a lack of economic stability, these small discussions on online platforms allow Myanmar netizens to reimagine a Myanmar that would be aligned with the democratic expectations that they hold. This does not lead to direct change; instead, it is an investment towards an inclusive society that is pivotal to establishing the foundations of order and peace.

Last, the prospect of change in Myanmar is higher from internally driven causes than externally imposed mechanisms. This argument is a challenge to the vast solutions taken by ASEAN to establish change in Myanmar. As a regional organization founded in 1976 and taking upon non-interference and non-intervention norms, ASEAN has been criticized for its passive action in the series of human rights violations conducted by the Tatmadaw for decades. This criticism was strong when ASEAN was not able to devise a proper response towards the Rohingya people (Jati, 2017; Limsiritong, 2018). So, when the military coup took place in 2021, it was in ASEAN’s interest to take decisive action in order to counter possible criticisms to reoccur. The Five Point Consensus (5PCS) was concluded several months after the coup. It called for an immediate cease of violence in the state, imposed the mechanism to allow humanitarian aid to enter the nation, and called to access all stakeholders in the conflict for mediating purposes (Chappell and Diaz, 2021; HRW, 2022; APHR, 2023; Muhammad et al., 2023). This was not taken lightly by the military rule of Myanmar. This ultimately led to a negotiation stalemate due to the inability of ASEAN to access those imprisoned by the Tatmadaw and attacks conducted towards the humanitarian aid entering the state (which the Tatmadaw declared the attacks perpetrated by terrorist organizations) (HRW, 2022). A regional solution to the crisis in Myanmar has never been able to generate an ideal state of affairs. This is because, as an authoritarian ruler, the Tatmadaw is sensitive towards actions that interfere with what they perceive is pivotal in running the country and has been the basis for continued actions consistent with legislative opportunism.

Nevertheless, the online movements in Myanmar face considerable challenges. The long-term presence of digital activism could fade, and significant obstacles could be faced due to several factors. Internally, if the youth starts to be disinterested in the movements, the liberation technology used since 2021 can quickly disappear and become insignificant. Suppose the Tatmadaw raises its awareness of the movements and decides to take more online platform restrictions and impose censorship on the media used by the online movements. In that case, the results of this will not be as favorable as what we saw as the result of the 2008 Saffron Revolution. Furthermore, externally, the international fatigue towards their concerns about the Myanmar crisis can also negatively impact the movement as locals in Myanmar would not have a target to voice their opinions and concerns.

## Data Availability

The original contributions presented in the study are included in the article/supplementary material, further inquiries can be directed to the corresponding author.
